# Medical Opinion and Movement

**Published:** 1908-09-12

**Authors:** 


### Medical Opinion and Movement.

An interesting discussion was called forth at the latest meeting of the Munich Society for the Study of Diseases in Children by a paper in which Dr. Moro lauded the use of vegetable soups in the treatment of assimilative disorders of children. Moro advises the exhibition of a carefully prepared carrot soup, and in the discussion it was pointed out that this was no novelty, since von Trumpp had already advised the use of vegetable soups in the treatment of digestive disturbances. Professor Oppenheim was inclined to ascribe the improvement in the author's cases to the saline ingredients, and stated that he had obtained equally satisfactory results with a veal broth. In cases of emaciation and extreme loss of fluid, vegetable soups are to be recommended, and it is possible that the vegetable element, owing to the cellulose particles, causes a slight intestinal irritation. Professor Pfaundler agreed with the author that such soups, carefully prepared, were a valuable addition to the diet, but thought it was not possible to decide whether they were merely irritative or whether they were really assimilated. He pointed out the importance of the addition of cellulose to the diet in cases of mal-assimilation, as in diabetes. Other speakers instanced equally good results following the addition of vegetable soups to the usual diet. Trumpp stated that he had used vegetable soups and fruit juice in the treatment of cases of chronic digestive disorders with the best results; in acute cases he had seen no great benefit following their administration.

Muller, of Rostock, following the Russian pioneers in this branch of surgery, has employed joint transplantation with satisfactory results in two cases. The first patient had a double-wrist deformity due to total congenital absence of the radius, and this was remedied by transplanting a portion of tibia with periosteum, articular surface, and epiphysis, and putting the hand in a plaster bandage in an over-corrected position. In the second case, in which there was a congenital deformity owing to absence of the lower third of the ulna, a similar portion of tibia was transplanted into the length of ulna remaining. Both cases healed by primary union, and in both the defect was remedied, giving a good result. Radiograms taken at different periods after the operation showed that the implanted bones were steadily progressing in size. This growth was not confined to the breadth of the implanted piece of bone, as is the case when a periosteum-covered fragment is transplanted, but was observable also at the extremities, so that there was every reason to hope that the cases were really cured, and that in one a new radius and in the other a new ulna had been formed. These results agree with what has been reported by other surgeons, and it seems possible that in the near future joint transplantation will become a fully legitimate operation in surgery. At present, however, the practical difficulties are still very great, and the technique of the operation will have to be considerably modified before the method becomes popular.

Von Mangold reports two cases of successful complete splenectomy for enlarged spleen, in the one case of cystic nature, and in the other due to myeloid degeneration. The operation is by no means new, for the first complete splenectomy was done as early as the seventeenth century, but the indications for the operation are still vague, and full reports of all cases in which it has been performed are to be welcomed. Both von Mangold's patients were women past fifty, and in the second case the patient had suffered for two years from malaria. The pathology in this case was obscure; the author is inclined to believe that the condition was one of "Banti's disease in its first stages." In the case of the cystic spleen the condition was typically one of the so-called cystoma papilliforme multiloculare, the cysts being clothed with a thick layer of cylindrical epithelium. Its ætiology is quite obscure; possibly it may be due to embryological causes. Clinically both cases showed an enormous enlargement of the spleen, which was chiefly directed downwards and inwards, and in neither case was there any leucocytosis, or indeed any marked alteration in the blood-counts which were systematically undertaken from time to time. In the discussion that followed the demonstration of these two patients before the Dresden Medical Society, Schmosl suggested that the second case may possibly belong to the group of atypical leukæmias, such as Nauwerk described. In these a condition of osteosclerosis is present, and Schmosl has himself had occasion to observe a case in which a patient died from severe intra-peritoneal hæmorrhage following herniotomy. In this case the condition of the spleen was very similar, a myeloid degeneration being the main feature present. In addition, however, there was marked osteosclerosis, all the spongy bone tissue being transformed into hard, compact bone. These cases of osteosclerotic leukæmia are very rare and have not been seriously studied. Clinically they show an enlargement of the spleen combined with a moderate leucocytosis and with a tendency to hæmorrhages, while the patients are in the majority of cases considerably past middle age.

At the German Otological Congress held at Heidelberg this year, Professor Wanner read an interesting paper on the otological disturbances due to congenital syphilis. Bezold found that out of 233 cases of acquired deaf-mutism more than 5 per cent. were due to congenital syphilis. In such cases the deafness appears between the eighth and tenth years. Wanner has followed up these investigations and finds that in the majority of cases the deafness occurs suddenly, either between the seventh and ninth or between the eleventh and thirteenth years, though in a few cases the patients have reached the twentieth year before the difficulty in hearing becomes manifest. Usually the ear disturbances are preceded by eye disorders, such as keratitis or iritis, while in nearly all cases well-marked signs of congenital syphilis exist. The diagnosis is often difficult, while the prognosis is grave as regards permanent cure. Rapid anti-syphilitic treatment is recommended, and the author has found the best results follow the exhibition of iodide in large quantities and inunctions. More important than the symptomatic treatment, however, is to prevent the patients from becoming dumb. Usually, when the ear symptoms are manifest the child has learned to speak, but in many cases not well, and owing to the total deafness there is grave danger that in such cases total loss of speech may result. The author, therefore, advises that such patients should be promptly taken in hand by competent deaf and dumb instructors and re-educated so as to be able to follow the lip movements. This re-education is less difficult with such children than with congenitally deaf and dumb patients, but it must none the less be carried out methodically and perseveringly, even though the anti-syphilitic treatment appears to improve the hearing. Relapses are not infrequent, and in a few cases the author has seen the deafness rapidly increasing under iodide treatment.

---

Dr. P. Clennell Fenwick, of Christchurch, New Zealand, has reported upon a further use he has found for the treatment by zinc ionisation, namely in cases of chronic urethritis. He gives details of a few cases in which the urethritis was of long standing, and had not yielded to all other forms of treatment, but quickly cleared up after two or three applications. The technique is quite simple. A cannula is passed up the urethra, and a long straight probe wrapped in fine lint, which has been soaked with a two per cent. solution of zinc sulphate, is passed through the cannula. The cannula is then withdrawn and the probe left in position in the urethra. The projecting end of the probe is connected with the positive pole of a constant current battery, and a large negative pad is placed under the patient's loins. Two to three milliamperes of current are then passed through the circuit for about ten minutes. The current only causes a slight tingling. The operation should be repeated every few days if necessary. In one case three sittings were sufficient to effect a cure, in another, five treatments were necessary. The canal frequently contracts on the probe so that care must be exercised in withdrawing it. Examined with a urethroscope immediately after treatment, the mucous membrane of the urethra is seen to have a glistening white appearance. As an alternative method, if the urethral canal is too small to take the probe with its wrappings, the canal may be filled with the zinc sulphate solution, and the corona constricted by a thin india-rubber cord. Small straight electrodes are then fixed above and below the penis by india-rubber bands, and a constant current passed as before.

---

The transplantation of tissues is a branch of surgery of peculiar interest, and one which has made rapid progress in recent years, owing to the greater certainty with which such operations can be devised and carried out under aseptic methods. Erich Lexer has recently reported some notable successes he has obtained in the transplantation of bone and cartilage for the treatment of ankylosed joints. The treatment of bony ankylosis by osteotomy, or by more extensive resection, generally results in recurrence of the ankylosis, or in the production of a flail joint. The interposition of soft parts—flaps of muscle, tendon, etc., have given somewhat better results. Weglowski made a distinct advance by transplanting plates of rib cartilage after partial resection of an ankylosed elbow. He was able to demonstrate that these grafts grew in place, shaped themselves to the joint surface, and provided a fair functional result. More recently Buchmann has obtained satisfactory results in two cases, by transplanting the first metatarsophalangeal joint after resection of ankylosed elbow joints. Lexer's cases are, however, more remarkable still. In two cases of ankylosed knee-joints he has implanted the whole joint, consisting of the epiphyses of the femur and tibia with semilunar cartilages and crucial ligaments, obtained from amputated limbs. The results are reported to be excellent. In a third case the upper end of a tibia was grafted from an amputated limb to replace a corresponding piece of bone removed for sarcoma. In a fourth case the lower end of the femur was substituted for the upper end of a humerus, also removed for sarcoma, one condyle was shaped to articulate into the glenoid fossa, and a piece of fibula was used as an intermedullary splint to unite the graft with the shaft of the humerus. The author has also substituted a toe phalanx for the proximal phalanx of a girl's hand, removed for enchondroma. Such wholesale transplantations successfully performed are very encouraging for further work on the same lines.

---

Although the *x*-rays have failed to fulfil the highest expectations that were formed by early enthusiasts in the treatment of malignant growths, their action has been shown to be so far effective and destructive of the carcinomatous tissue that surgeons frequently recommend a course of treatment after operative interference with a view to effect a destruction of any remnants and so prevent recurrence. De Keating-Hart, of Marseilles, however, dissatisfied with the results obtained by the *x*-rays, has been experimenting with the spark rays of d'Arsonval's high frequency currents, and has devised a method of treatment by which he combines surgical removal of the tumour with the application of these rays. He finds that the high frequency currents with very high tension cause a necrosis of the malignant tissue, and at the same time stimulate the surrounding normal tissue to absorb the necrosed parts and to form a firm scar. The usual d'Arsonval apparatus is used and the maximum tension is estimated at 250,000 to 300,000 volts, while the current itself is quite weak. To obviate burning, hollow electrodes are used through which a stream of carbonic acid gas is passed from a cylinder. The application is painful, and must be carried out under anæsthesia. The tumour or the line of incision is first sparked for a few minutes, then the operation of removal is carried out in the usual way, and the wound is then sparked again for about 45 minutes. The skin and wound surfaces become ischæmic, but no actual scab is formed. Benckiser and Krumm of Karlsruhe have recently witnessed this method of treatment carried out by De Keating-Hart, and report favourably upon it. It is still on its trial, but so far some very successful results have been obtained both by using the sparking rays alone and also, as mentioned above, in combination with the usual surgical operations.

---

THE surgical value of injections of nuclein as a means of producing a hyperleucocytosis and so increasing the resisting powers of the organism against infection is discussed by Dr. W. F. Waugh, of Chicago, in a recent issue of the *International Journal of Surgery*. Miyake has shown that by subcutaneous injections of nuclein the number of leucocytes in the blood may be increased to eight times the normal, and by experimenting on animals he found that the resistance of the peritoneum against infective germs was enhanced to the extent of sixteen to twenty times its ordinary power. Acting upon these experimental results, Von Mikulicz and Renner have used injections of nuclein in a number of cases with a view to increasing the resisting power of the peritoneum against infection by the bacillus coli, and report favourably of the treatment. Paukow has administered nuclein prophylactically in a large number of gynæcological operations and is favourably impressed with the results, and Hanner, of Breslau, also reports favourably of its prophylactic value, and is disposed to attribute the decline in post-operative mortality, amounting to 20 per cent. in a year, in part, at least, to the nuclein treatment. The question arises whether the favourable results are to be attributed to the nuclein itself or to the hyperleucocytosis which it occasions. The question is important, as experiments show that Abbott's standard solution of nuclein administered hypodermically up to doses of 60 minims per diem cause a constant proportional increase in the number and activity of the leucocytes, but when this dose is exceeded an enormous fall in the leucocyte count ensues. Dr. Waugh calls attention in this connection to the fact that marked improvement in cases of carcinoma has been reported by hypodermic injections of nuclein in doses of half an ounce per diem, and suggests that the improvement may in such cases be due to the destruction of leucocytes which such doses would occasion.

---

IT is always interesting, and sometimes instructive, to hear the opinions of strangers on our institutions and customs, and when they are expressed in such guarded and temperate terms as Dr. Schütt uses in dealing with medical life in Australia they make really interesting reading. Dr. Schütt has practised for many years in Melbourne, and in a recent number of the *Münchner Medizinische Wochenschrift* he gives his impressions of medical life in that part of the Empire for the benefit of his German colleagues who may be wishful to emigrate to Australia, there to seek their fortunes. According to him, it is easy for the practitioner to make, if not a fortune, then at least an appreciable competence, but he adds a warning to the effect that this El Dorado, where a visit is paid for at the rate of a guinea and a simple surgical operation brings in 200 marks, is an expensive land to live in. He rightly has a dig at the absurd "reciprocity regulations" which forbid a practitioner who possesses a German M.D. from practising in a country where the man with a qualification from a local university is allowed his license, and details a few of the complications which have resulted from such a ruling. The picture he draws of the social conditions is not an attractive one, and we can only hope that it is very much overcoloured. According to him, Australia has civilisation but comparatively no culture. Australian colleagues are friendly and fraternal in the extreme, but the German doctor is nevertheless "feared and hated." We can only regret that Dr. Schütt has not found his dinner parties more attractive than they appear to have been, and we leave it to our Australian contemporary to press home the inevitable *tu quoque* which his remarks on local social conditions calls forth. For all that we shall look forward with interest to what he has to say on the subject of medical education in the Australian Commonwealth, and if the future article he promises on this subject is as interesting as his first letter it ought to be instructive reading.

---

PROFESSOR STIEDA read a paper on acute appendicitis due to the presence of actual foreign bodies in the lumen of the appendix, before a recent meeting of the Medical Society of Halle. His case was that of a girl of twenty who was operated upon at the surgical clinic for acute appendicitis. At the operation a small piece of lead, obviously a bit of pheasant shot, was found in the lumen of the appendix, which was abnormally wide. The appendix itself was only slightly inflamed, and a point of interest in the case was the fact that the patient belonged to an "appendicular family," three other members of whom had suffered from appendicitis. Stieda is inclined to believe that such cases are very rare, as out of 550 cases of acute appendicitis an actual foreign body was found in only two—in one case it proved to be a grape seed and in another a bit of lead. This is not, however, such a rare observation, as most museums show specimens of actual foreign bodies blocking the lumen of the tube. In one case which came under our own observation the appendix was literally stuffed with grains of shot, no less than forty-eight of which were removed from its lumen after the operation. Clinically, such cases are of interest from a prognostic and pathological point of view. Although the patient may show signs of acute appendicitis in an exaggerated degree, the organ is usually found to be but little affected at the time of operation, and the condition is really one of irritative catarrhal inflammation. The patients usually recover promptly, and it remains still to be proved that the presence of the foreign body was the cause of the trouble. Generally, as in Stieda's case, the lumen in such cases is inordinately wide, and in the case mentioned above the accumulation of shot was obviously gradual and not sudden. Such patients suffer from intermittent pains in the right iliac fossa, and it is only reasonable to believe that the presence of a foreign body is not the immediate cause of the attack, but merely irritates an already diseased organ and so leads to the sudden and acute onset that brings the case to the operating table.

